# The Effect of Stereocomplexation and Crystallinity on the Degradation of Polylactide Nanoparticles

**DOI:** 10.3390/nano14050440

**Published:** 2024-02-28

**Authors:** Chuan Yin, Jenny Hemstedt, Karl Scheuer, Maja Struczyńska, Christine Weber, Ulrich S. Schubert, Jörg Bossert, Klaus D. Jandt

**Affiliations:** 1 Chair of Material Science (CMS), Otto Schott Institute for Materials Research (OSIM), Friedrich Schiller University Jena, Löbdergraben 32, 07743 Jena, Germany; chuan.yin@uni-jena.de (C.Y.); hemstedt99@gmail.com (J.H.); karlscheuer31@gmail.com (K.S.); maja.struczynska@uni-jena.de (M.S.); joerg.bossert@uni-jena.de (J.B.); 2 Jena School for Microbial Communication (JSMC), Neugasse 23, 07743 Jena, Germany; 3 Institute of Organic Chemistry and Macromolecular Chemistry (IOMC), Friedrich Schiller University Jena, Humboldtstrasse 10, 07743 Jena, Germany; christine.weber@uni-jena.de (C.W.); ulrich.schubert@uni-jena.de (U.S.S.)

**Keywords:** polymer nanoparticles, poly l-lactide, poly d-lactide, drug delivery, degradation, stereocomplex, nanostiffness

## Abstract

Polymeric nanoparticles (PNPs) are frequently researched and used in drug delivery. The degradation of PNPs is highly dependent on various properties, such as polymer chemical structure, size, crystallinity, and melting temperature. Hence, a precise understanding of PNP degradation behavior is essential for optimizing the system. This study focused on enzymatic hydrolysis as a degradation mechanism by investigation of the degradation of PNP with various crystallinities. The aliphatic polyester polylactide ([C_3_H_4_O_2_]n, PLA) was used as two chiral forms, poly l-lactide (PlLA) and poly d-lactide (PdLA), and formed a unique crystalline stereocomplex (SC). PNPs were prepared via a nanoprecipitation method. In order to further control the crystallinity and melting temperatures of the SC, the polymer poly(3-ethylglycolide) [C_6_H_8_O_4_]n (PEtGly) was synthesized. Our investigation shows that the PNP degradation can be controlled by various chemical structures, crystallinity and stereocomplexation. The influence of proteinase K on PNP degradation was also discussed in this research. AFM did not reveal any changes within the first 24 h but indicated accelerated degradation after 7 days when higher EtGly content was present, implying that lower crystallinity renders the particles more susceptible to hydrolysis. QCM-D exhibited reduced enzyme adsorption and a slower degradation rate in SC-PNPs with lower EtGly contents and higher crystallinities. A more in-depth analysis of the degradation process unveiled that QCM-D detected rapid degradation from the outset, whereas AFM exhibited delayed changes of degradation. The knowledge gained in this work is useful for the design and creation of advanced PNPs with enhanced structures and properties.

## 1. Introduction

The development of drug delivery systems for targeted drug applications dates back to as early as the 1950s [1]. Over the last several decades, this technology has undergone continuous improvements [1,2]. The foremost benefit of drug delivery systems is precise control over the active ingredient release into the human body, which often results in reducing the required dosage of the active ingredients. Consequently, this may prevent the common side effects associated with commercial medication [3]. The drug release system involves three main steps: drug diffusion, chemical or enzymatic reactions that lead to system degradation, and solvent activation. The steps can be combined for the desired release type to provide precise control over time and location [1,2,3,4].

Polymeric nanoparticles (PNPs) are frequently researched and used in drug delivery, especially for the targeted treatment of tumor or cancer cells through a targeting process [5]. In this process, specific ligands are attached to the PNP’s shells to guide the drug to its desired site of action [6]. The biocompatibility of the polymers and their degradation products are crucial for drug delivery. Drug delivery polymers must be biodegradable to avoid potential harm. The retention of PNPs in the body for extended periods is generally undesirable [7]. The complex chemical nature of body fluids, variability in the polymer material, and the variability associated with biological systems (e.g., the rate of metabolism of the host) make the overall degradation of biomaterials a rather complex process. Biological processes and components such as host response, cell activities, tissue regeneration, and enzymatic reactions all contribute to the degradation [8,9]. Furthermore, the degradation of PNPs is highly dependent on various properties such as polymer chemical structure, size, crystallinity, and melting temperature. Hence, a precise understanding of PNP degradation behavior is essential for optimizing the system. However, the complex interaction between physical and chemical properties makes it extremely challenging to completely separate the individual properties of PNPs and determine their influence on degradation.

PNP degradation can occur via surface erosion or bulk erosion, depending on the polymer hydrophobicity and water transport ability [10]. Polymers with enhanced hydrophobicity primarily undergo surface erosion, where water penetration is slow or negligible, causing chain and bond cleavage starting from the exterior. In contrast, moderately hydrophobic polymers (e.g., polylactides, polyglycolides, and poly(lactide-co-glycolide)) undergo bulk erosion, as water rapidly diffuses within the material, it causes even chain cleavage throughout the volume [11,12]. In enzymatic hydrolysis, factors like enzyme concentration and activity become relevant. These enzymes serve as natural biocatalysts, speeding up hydrolysis [11,12].

In addition to the hydrophobicity of the polymer, the degradation behavior is also determined by other properties such as the crystallinity, melting temperature, and molar mass [12,13,14]. According to Karavelidis et al., the lower the melting temperature, glass transition temperature, and crystallinity are, the faster the active ingredient release occurs [15,16]. Also, they found that the amorphous areas degraded faster than the crystalline areas. However, when investigating the predominantly hydrophobic polymers [15,16], the hydrophilic–hydrophobic balance (HHB) was not kept constant [17]. According to Scheuer et al. and Bandelli et al., the HHB of a molecule represents an important and decisive influence on the release of the drug [18,19,20]. The more hydrophilic the polymer is, the higher the degradation rate becomes due to the increased accessibility of water to the chemical bonds, resulting in faster hydrolysis [10,11]. However, the release of the active ingredient is not only determined by the degradation rate of the polymer but also by the interaction between the active ingredient and the polymer and the hydrophilicity of the drug [16]. Currently, it is still impossible to completely decouple all these variable parameters from each other; the influences of individual parameters on the degradation behavior have not yet been sufficiently and separately investigated. To exclude the influence of the increasing HHB value on the degradation, we worked with polymers that have a constant HHB despite variable crystallinities and melting temperatures.

**Figure 1:**
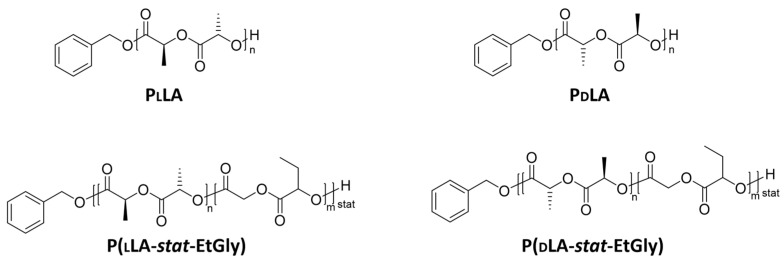
Chemical structures of PlLA, PdLA, P(lLA-*stat*-EtGly), and P(dLA-*stat*-EtGly).

This study focused on enzymatic hydrolysis as a degradation mechanism by investigation of the degradation of PNPs with various crystallinities. Compared with previous studies [18,19,20,21,22,23], polymers with a constant level of hydrophilicity were used. The aliphatic polyester used in this current study, polylactide ([C_6_H_8_O_4_]_n_, PLA), undergoes biodegradation via hydrolysis into non-toxic byproducts such as lactic acid and primarily degrades through bulk erosion [18,19,20]. The two chiral forms, poly l-lactide (PLLA) and poly d-lactide (PdLA), used in this study are shown in Figure 1 with their chemical structures and spatial orientations. When both forms coexisted, they formed a stereocomplex (SC), a unique crystalline structure resulting from stereoselective interactions. Compared to pure PlLA or PdLA, the physical properties are altered in SC, which feature for example a higher melting temperature and a stronger resistance to hydrolysis [18,19,24]. PNPs were prepared via Fessi et al.’s nanoprecipitation method [25], which is mainly used for encapsulating hydrophobic drugs [24]. In order to further control the crystallinity and melting temperatures of the SC, the polymer poly(3-ethylglycolide) [C_6_H_8_O_4_]_n_ (PEtGly) was synthesized [18,20]. The monomers ethylglycolide (EtGly), lLA, and dLA each have the same ratio of hydrophilic to hydrophobic groups, so the associated polymers all have the same HHB [20]. EtGly can form statistical copolymers with lLA and dLA via ring-opening polymerization [20]. The longer side chain in EtGly represents a steric hindrance and inhibits crystallization. In addition, the racemic stereocenter in EtGly can also contribute to this effect [18]. In consequence, a higher EtGly content results in lower crystallinity, which changes the stiffness and elastic modulus of the PNP [18]. This is why samples containing increased fractions of EtGly feature lower stiffness, reduced modulus of elasticity, and reduced melting temperature [18]. To enzymatically catalyze the hydrolysis process, the enzyme proteinase K was used based on the study of Yamashita et al. [26]. It was shown that it is one of the potential enzymes involved in the degradation of PlLA [27] and can be found in all living organisms [28]. PLA degradation occurs via the simple hydrolysis of ester bonds [10,11]. It is reported that this enzyme preferentially hydrolyzes PlLA instead of PdLA. A higher proportion of PdLA as well as an increased crystallinity may thus decelerate degradation [28].

Our investigation shows that PNP degradation can be controlled by various chemical structures, crystallinity, and stereocomplexation. The influence of proteinase K on PNP degradation is also discussed in this research.

## 2. Materials and Methods

### 2.1. Materials

PlLA and PdLA were synthesized by Bandelli et al. [20] through ring-opening polymerization. The cationic polymer poly(2-(*N*,*N*-dimethylamino)ethyl methacrylate) (PDMAEMA) was purchased from Sigma-Aldrich Chemie GmbH (Munich, Germany) to impart positive charges to the substrate surface for later functionalization. Proteinase K for degradation evaluation was purchased from Promega Corporation (Madison, WI, USA) at a concentration of 0.1 mg/mL and was stored at room temperature in a buffer solution containing 10 mM Tris-HCl and 1 mM calcium chloride. Sodium dodecyl sulfate (SDS) solution (Promega Corporation, Madison, WI, USA), used to increase enzyme activities, was stored at room temperature in the same buffer solution as the proteinase K.

### 2.2. Preparation of SC

Stereocomplexes comprising the copolymers P(lLA-*stat*-EtGly) and P(dLA-*stat*-EtGly) with 5, 10, and 20 mol% of the lactide isomer ethylglycolide (EtGly) were prepared based on the method used by Scheuer et al. [18]. The selected characterization is shown in Table 1. The statistical copolymers were used for the formulation of SCs (50/50 l-lactide/d-lactide feeds, respectively, were used). In brief, PlLA- and PdLA-based materials were mixed 1:1 (wt%) and dissolved in tetrahydrofuran (THF) with a concentration of 1.0 mg/mL, then kept for 7 days for self-assembly and formation of the SC.

### 2.3. Preparation of PNPs via a Nanoprecipitation Process

PNPs of various crystallinities were prepared using the nanoprecipitation technique that was first mentioned by Fessi et al. [25] and is based on the Marangoni effect. The process can be divided into four steps. (1) Preparation of a liquid phase (oil phase) in which the polymer and the active ingredient are readily soluble. (2) Preparation of another liquid phase (water phase) in which the polymer and the active ingredient are only sparsely soluble or not soluble at all. The first phase should be readily miscible with the second phase. (3) Mixing the two phases by dropping the oil phase into the water phase under constant stirring. Then, the solvent in the oil phase may quickly diffuse into the water phase, so that nanoparticles form instantaneously in the resulting colloidal suspension [29]. (4) Removal of the oil phase, for example by evaporation.

**Figure 2:**
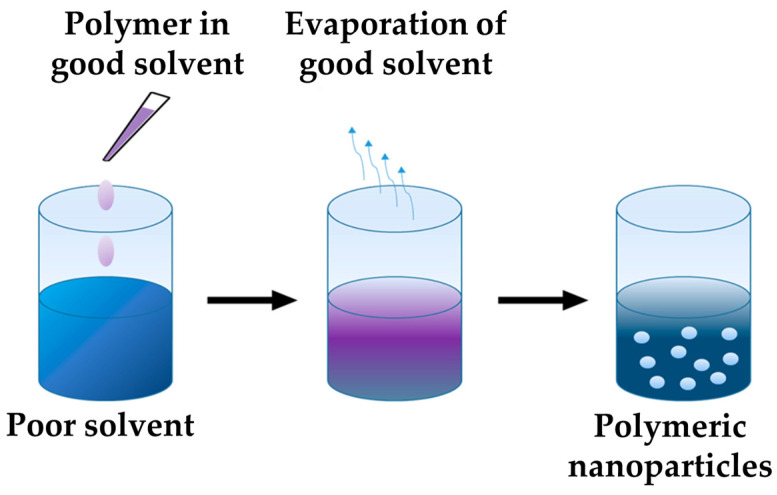
Schematic illustration of the nanoprecipitation process (good solvent here refers to a solvent with a comparably higher evaporation rate than the poor solvent).

In this study, THF was used as the oil phase to dissolve the polymer. The solution was dropped into Milli-Q Water (MQW) at a constant rate of 200 µL/min by using an LA30 syringe pump (Landgraf Laborsysteme HLL, Langenhagen, Germany) while stirring continuously using a magnetic stir bar at a frequency of 1000 rpm. The process is shown schematically in Figure 2. Disposable syringes (3 mL NORM-JECT, Henke Sass Wolf, Tuttlingen, Germany) and disposable needles (100 STERICAN, B. Braun, Melsungen, Germany) were used in this process. An amount of 1.0 mL THF solution was dropped into 1.4 mL MQW, then the suspension was stirred continuously and left in the fume hood for three hours for solvent evaporation. During this time, both the THF and MQW evaporated, leaving behind 1.0 mL of PNPs that were dissolved in MQW and maintained a polymer concentration of 1.0 mg/mL. The prepared PNPs were expected to have a size distribution with a hydrodynamic radius (R_h_) of around 100 nm, displaying a monomodal size distribution [18]. A narrow size distribution and uniform particle size is preferred to exclude particle size as a potential influencing factor in degradation.

To obtain SC-PNPs, the self-assembly of SC formation in THF was stopped after 7 days [18]. Since THF is a suitable solvent for homochiral PlLA and PdLA, partially crystalline compounds cannot form before nanoprecipitation in solutions containing only one chiral form. Consequently, PNPs formed from a single PLA exhibit amorphous properties without any stereocomplex formation. Therefore, no difference in the properties is expected here, even with different EtGly amounts in the polymer.

### 2.4. Immobilization of PNPs by Surface Functionalization

To investigate the PNPs by atomic force microscopy (AFM) and quartz crystal microbalance (QCM), PNPs were immobilized on the substrate. Therefore, silicon wafers for AFM and gold chips for QCM were functionalized after cleaning.

Substrates were immersed in 10 mg/mL PDMAEMA aqueous solution for 15 min, followed by 10 to 15 s of water bath rinsing process 3 times. After surface functionalization, the PNPs were deposited in water onto the substrate and analyzed by AFM and QCM after drying [30]. 

### 2.5. Degradation Evaluation

To increase the enzyme activity, SDS was added to the enzyme solution with a proportion of 0.5 wt%. To initiate PNP degradation, each substrate was added to 2 mL of proteinase K solution and allowed to rest at 37 °C. The degradation process was stopped after 90, 180, 270, and 360 min and after 24 h by rinsing the substrate with a 40% ethanol solution to remove the enzyme from the particles.

To investigate the degradation behavior of the SC-PNPs, 6 Si substrates were functionalized and particles were deposited on them for all four particle suspensions (0, 5, 10, and 20 mol% EtGly). The samples were numbered from 01 to 06, and five out of the six samples were subjected to degradation for different durations. The degradation process was carried out at a temperature of 37 °C and an enzyme concentration of 0.1 mg/mL. The degradation time for the first four samples was gradually increased by 90 min, up to a total duration of six hours. Subsequently, the last sample was degraded for 24 h.

### 2.6. Dynamic Light Scattering (DLS) Measurement

To determine PNPs size, DLS measurements were carried out using a Zetasizer Nano ZS instrument (Malvern Instruments, Herrenberg, Germany) at 37 °C, with a wavelength of λ = 633 nm and a scattering angle of 173°. Disposable UV cuvettes makro (BRAND, Wertheim, Germany) were utilized for the measurements.

### 2.7. Atomic Force Microscopy (AFM) Measurement

AFM microscopy was used to determine the height and amount of PNPs before and after degradation. Images of the samples before and after degradation were obtained using the MultiMode AFM (Digital Instruments, Veeco, Santa Barbara, CA, USA) in a tapping mode. Conventional cantilevers made of silicon (model FESP-V2, Bruker, Santa Barbara, CA, USA), with a resonance frequency of 75 kHz in air and a spring constant of 2.8 N/m, were used. The PNPs’ heights were measured via cross section lines carried out by 100 particles per sample.

Due to the repulsive and attractive forces, the vibration amplitude and the phase change during the surface scanning [31]. While the amplitude image provides topographical information, the phase image contains information about the mechanical properties as a hard–soft contrast [31].

AFM was also employed to measure the mechanical properties of PNPs. The tip–sample interaction was monitored via the vertical cantilever deflection on a single contact point. Changes in the force between the tip and sample caused bending of the cantilever. Force–distance curves (FDCs) were recorded during tip approaching and retraction. Young’s modulus was determined from the slope of the force–distance curve in the contact region [31]. Here, FDCs were recorded using the JPK NanoWizard 4 AFM (JPK Instruments AG, Berlin, Germany) in contact mode. Conventional silicon cantilevers (model RTESPA-300, Bruker, Santa Barbara, CA, USA) with a resonance frequency of 300 kHz in air and a spring constant of 40 N/m were used. At each pixel, a measurement curve was recorded and subsequently analyzed using the associated JPK Data Processing software. Young’s modulus of the substrates was calculated by the Hertz model.

### 2.8. Quartz Crystal Microbalance with Dissipation (QCM-D)

To observe the PNP degradation in situ, measurements of mass change and dissipation were conducted via QCM-D on the Q-Sense E1 QCM-D instrument (Q-Sense, Gothenburg, Sweden). The substrates used were piezoelectric quartz crystals with gold electrodes (Au chip) obtained from Q-Sense (Gothenburg, Sweden) prior to a QCM-D chamber from KSV Instruments (Helsinki, Finland). QCM chips were exposed to proteinase K for a specific period of time. During the measurements, the chamber was maintained at a constant operating temperature of 37.0 ± 0.1 °C using a temperature controller from Oven Industries Inc. (Mechanicsburg, PA, USA). This temperature control ensured consistent conditions throughout the measurement process.

### 2.9. Statistical Analysis

In this study, a simple one-way ANOVA was conducted using Origin 8.1 software. The Dunn–Sidak mean comparison was employed at a significance level of *p* = 0.05 to assess the differences between the group means.

## 3. Results and Discussions

### 3.1. Particle Sizes

#### 3.1.1. Particle Sizes Measured Using DLS

**Figure 3:**
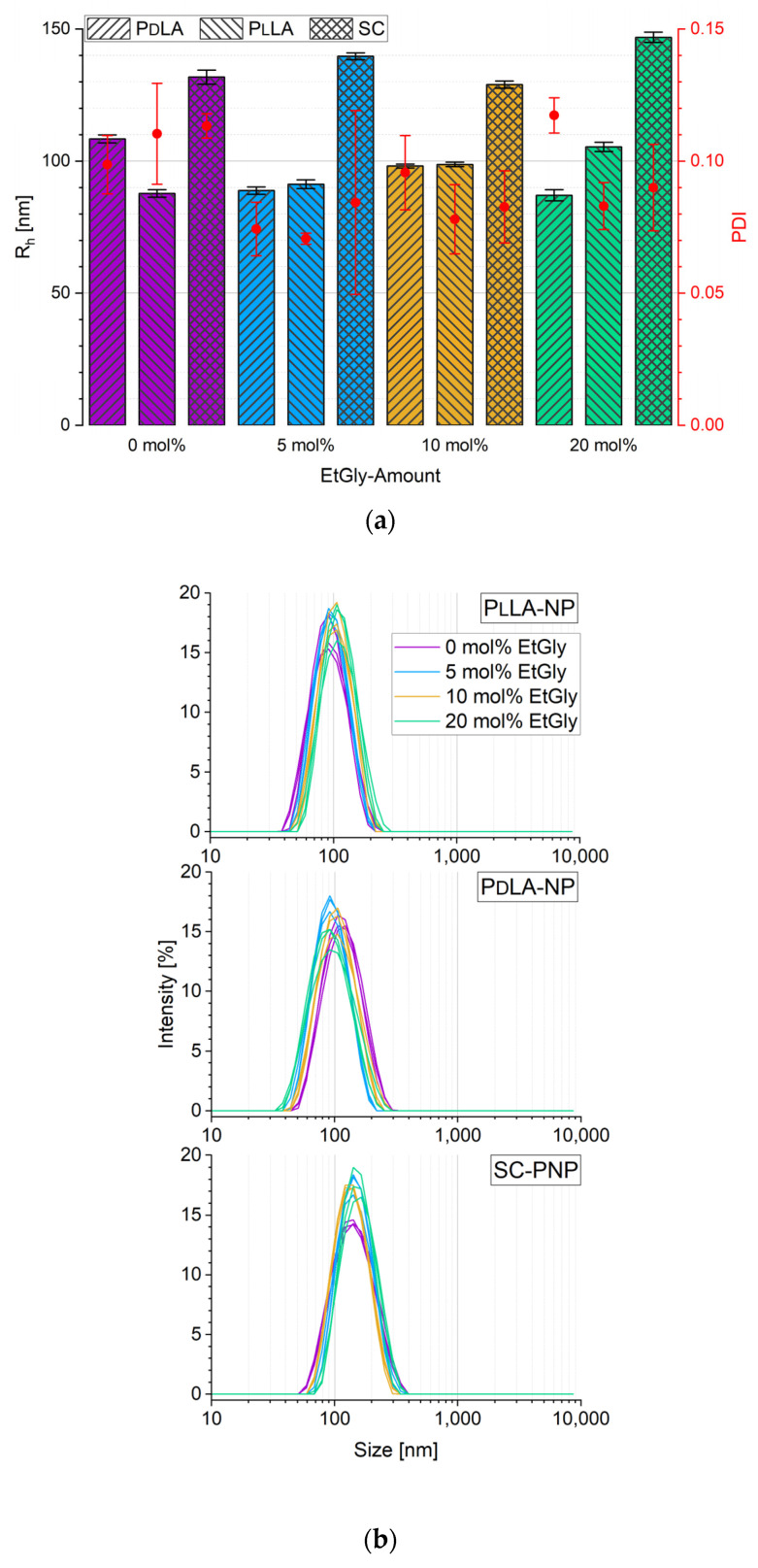
Hydrodynamic radius R_h_ (bars) and polydispersity index PDI (red dots) of the prepared PNPs with different EtGly amounts (**a**) and DLS curves (**b**) for all PNPs in suspension. The error bars indicate the standard deviation (SD) from the mean.

To exclude particle size as a factor influencing degradation, PNPs with a narrow size distribution were prepared by keeping constant parameters during nanoprecipitation. All PNPs in suspension were examined by DLS. The hydrodynamic radius (R_h_) and polydispersity index (PDI, the distribution of molecular mass in a specific polymer) values are shown in Figure 3a. A total of 30 measurements were conducted for each sample, and the average value with the corresponding standard deviation was calculated from these results. All measurement curves consistently exhibited a single peak, indicating that the size distribution of the samples can be considered monomodal. Exemplary curve profiles are shown in Figure 3b. It is noteworthy that the PNPs without stereocomplexation are significantly smaller than the SC-PNPs. The R_h_ of all SC-PNPs, regardless of the EtGly content, is 137 ± 7 nm (from 130 to 144 nm), which aligns with the desired target size for drug delivery [6]. However, without the prior formation of a SC, the R_h_ is only 98 ± 13 nm, close to the expected minimum size of 100 nm. Despite variations in PlLA or PdLA, as well as differences in the EtGly content, the size range of the PNPs remains consistently unchanged.

#### 3.1.2. Particle Sizes Measured Using AFM

**Figure 4:**
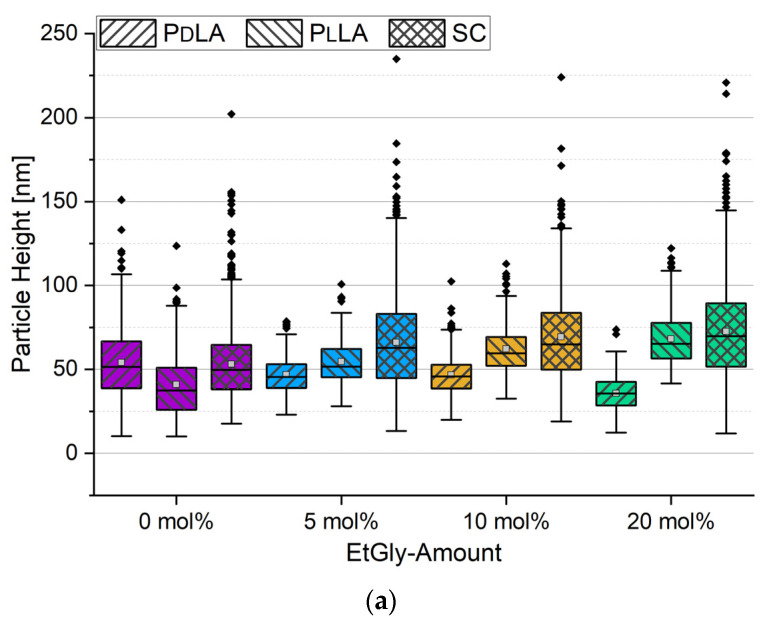

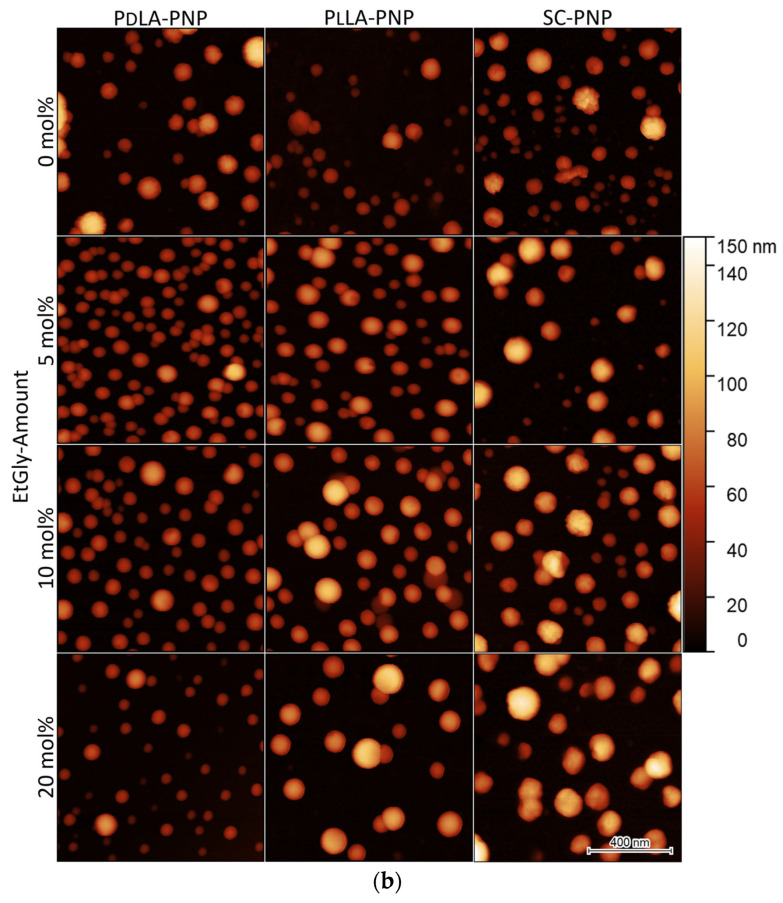
Particle heights of the prepared PNPs with different EtGly amounts determined by AFM (**a**) and representative AFM images of the PNPs on functionalized Si substrates (**b**). The height scale on the right side and the lateral scale in the bottom right image (400 nm) apply to all images.

The particle heights measured via AFM are compared with the results obtained from the DLS measurements (Figure 4a). A total of 100 measurements were taken to obtain a representative dataset for each type of PNP. By functionalizing the Si substrates with PDMAEMA, the particles tended to increase their contact area on the substrate, resulting in a flattened shape [30]. Consequently, the measured height was always smaller than the R_h_ determined by DLS [32]. The measured heights for the PNPs are shown in Figure 4. The middle 50% of the data is represented by the boxes. The antennas represent the remaining measurement data around the center, with their length limited to 1.5 times the box length. Data points further out are marked separately as outliers. The median as a solid line and the mean as a small square are additionally recorded in the box.

When comparing the positions of the means in Figure 4a with the height bars in Figure 3a, a clear agreement can be observed in the size trends of the particles among each other. However, the presence of long boxes and a large number of outliers suggests that the size distribution varies more than expected based on the low PDI values. This observation is consistent with the AFM images in Figure 4b. As a consequence of this observation, 100 particles were consistently evaluated to obtain a representative dataset and enable statistical analysis using ANOVA. Measuring a large sample aims to compensate for the randomness of the measurement areas on samples with non-ideally homogeneous particle distributions, as well as the general size variations of the particles. Therefore, the random sampling measurement can be considered representative of the entire sample.

#### 3.1.3. Degradation Evaluation of SC-PNPs

**Figure 5:**
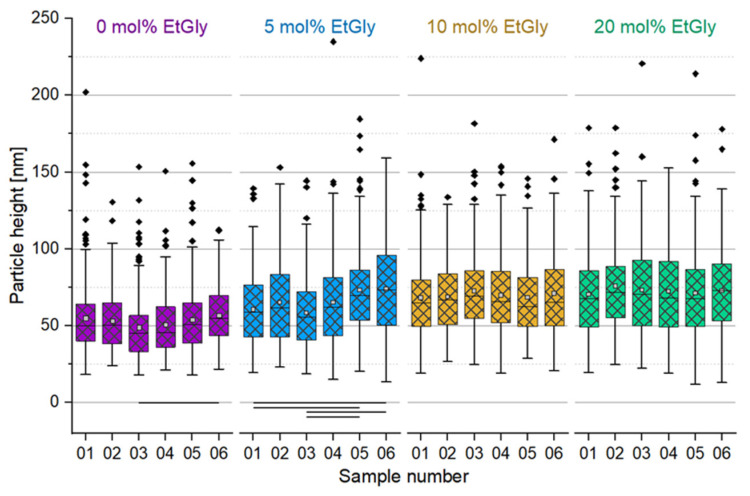
Particle heights of SC-PNP samples before degradation determined using AFM. Lines below bars indicate statistical differences in particle height in the group (*p* < 0.05).

An overview of the degradation times for the different PNPs, as measured using AFM can be found in Table 2. An exemplary AFM images before and after the degradation of SC-PNPs are presented in Figure S1. To ensure comparability among the different samples, the heights of the deposited particles on all substrates were measured prior to degradation and evaluated using a box plot. The initial situation is depicted in Figure 5. The six sets of samples, which correspond to each other and have the same EtGly content in the deposited particles, were compared to each other using ANOVA with the Dunn–Sidak test (*p* = 0.05). Statistically significant differences are marked with a connecting line below the boxes.

**Figure 6:**
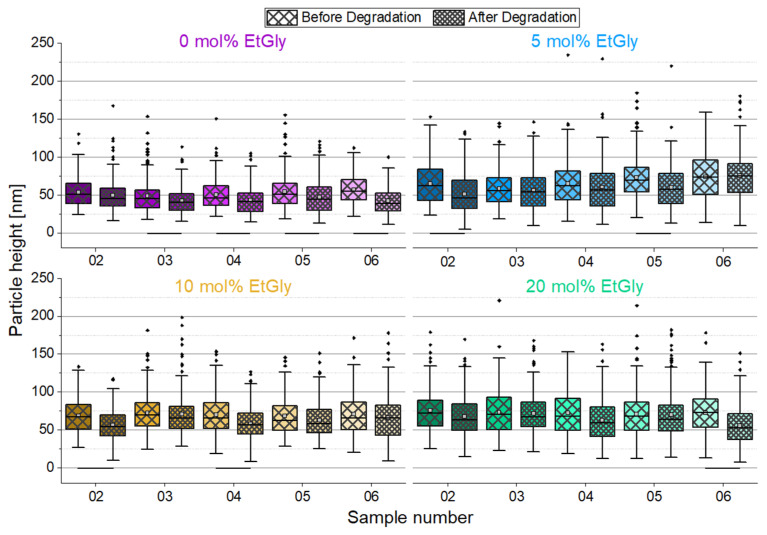
Particle heights of the SC-PNPs determined using AFM after degradation compared with the measured values before degradation. Lines below bars indicate statistical differences in particle height before and after degradation (*p* < 0.05).

Figure 5 shows relatively consistent particle size distributions across all substrates, with minor fluctuations. However, even before degradation, there are statistically significant differences among individual samples due to inherent variations in particle sizes. To account for these fluctuations, we directly compared the post-degradation average particle sizes to their initial states on the corresponding substrate, as shown in Figure 6. Our analysis indicates that particles tend to be slightly smaller after degradation, but not all cases show statistically significant differences. These differences do not follow a clear trend and appear to be influenced more by general size fluctuations. Additionally, there is no consistent evidence that particles decrease further in size after 24 h degradation compared to shorter times, especially when considering particle height only.

**Figure 7:**
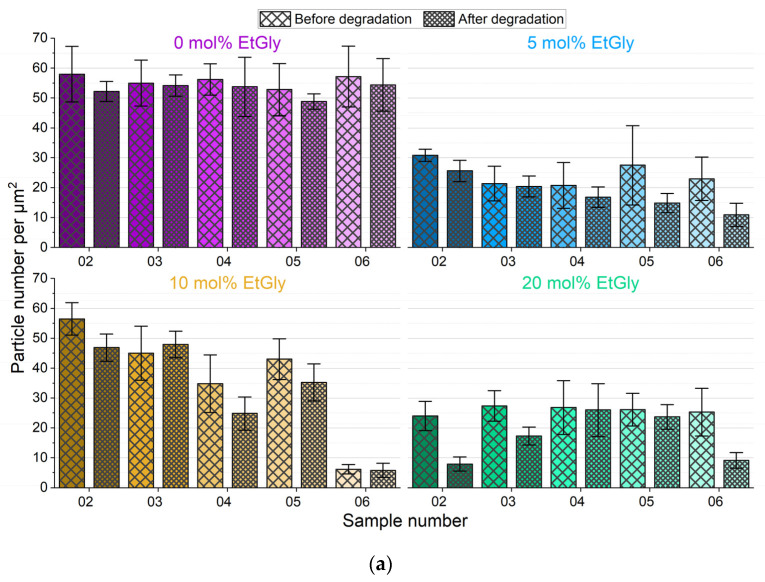

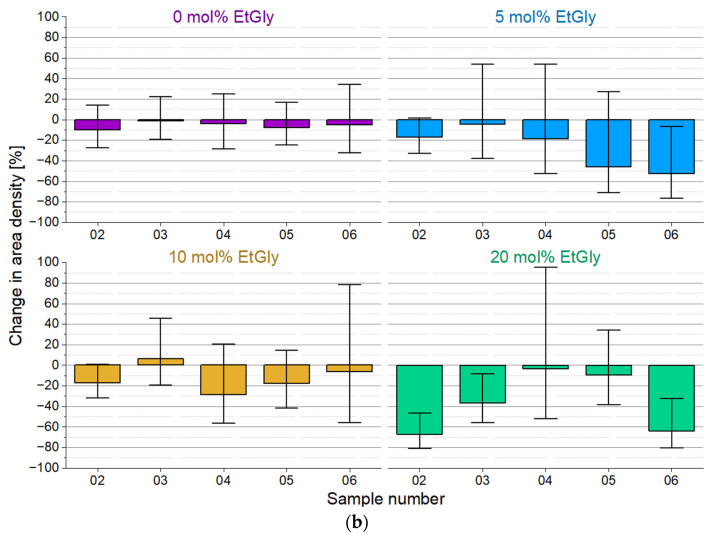
Area density of SC-PNPs before and after degradation on all samples determined using AFM (**a**) and percentage change in area density of SC-PNPs during degradation on all samples (**b**).

The degradation mechanism of SC-PNPs can be elucidated as potentially involving volumetric erosion, thereby resulting in minimal or negligible size reduction until complete dissolution and disintegration [18,33]. Therefore, in addition to the particle size discussed earlier, the coverage of the substrates by the particles as surface density (number of particles per square micrometer) was evaluated. For this purpose, the visible particles in the AFM images were counted and normalized to an area of 1 μm × 1 μm, considering the total area of all images. The absolute data of this counting analysis can be found in Figure 7a. To provide a more illustrative representation independent of the absolute surface density, the percentage change in surface density during degradation has been visualized in Figure 7b.

Counting the particles per unit area reveals a trend indicating that there are fewer particles on the samples after 1.5 h of degradation. The surface density decreased with increasing degradation time. However, it is also evident that the error bars of the standard deviation mostly encompass values in both directions around zero. Therefore, a definitive statement cannot be made at this point.

**Figure 8:**
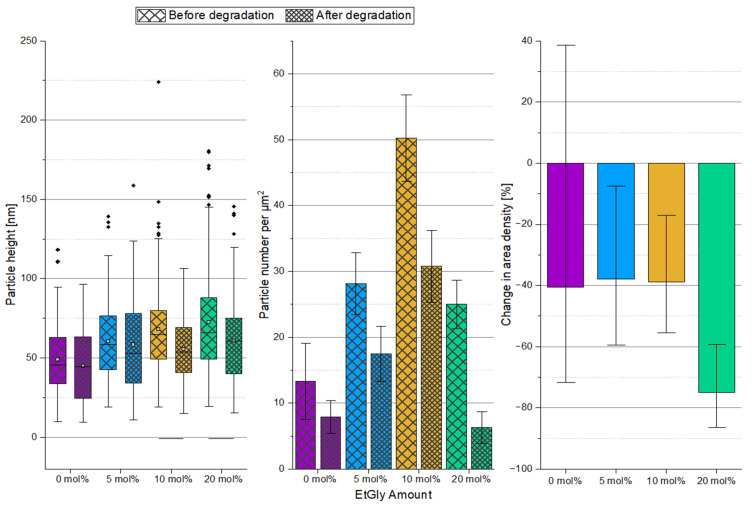
Particle heights (**left**) and area densities (**middle**) of the SC-PNPs determined via AFM before and after degradation for 7 days and the percentage change in area density (**right**) during degradation. Lines below bars indicate statistical differences in particle height in the group (*p* < 0.05).

To further investigate the SC-PNP degradation, an additional experiment was conducted. In this experiment, the enzyme concentration was increased by a factor of 10 to 1 mg/mL and SDS solution was added to the proteinase K. Furthermore, the degradation test duration was set to seven days. Subsequently, the particle heights and the surface densities of the particles before and after degradation were evaluated and graphically represented, as shown in Figure 8. The particle heights showed minimal changes after degradation, with only a statistically significant difference observed for EtGly contents of 10 and 20 mol%. However, when considering the surface density of particles before and after degradation, a clear trend can be observed. After degradation, there are significantly fewer particles present on the substrate compared to before. Furthermore, the proportion of disappearing particles increases with a higher EtGly content, which might be due to the lower degree of crystallinity in the polymer. Based on the findings, it can be concluded that the SC-PNPs degrade in the presence of the proteinase K and appear to disintegrate either without or with minimal prior loss of size.

**Figure 9:**
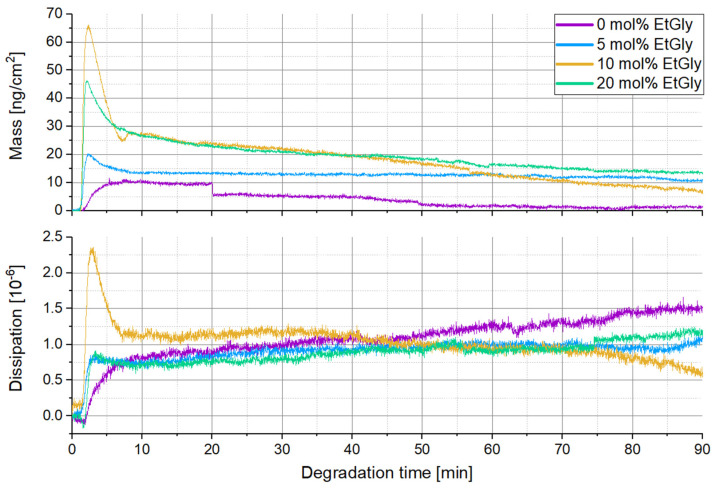
QCM-D measurement curves of SC-PNP degradation.

Finally, the degradation of SC-PNPs was also observed in situ using QCM-D measurements. QCM enables real-time monitoring of the degradation process. This means that the mass changes of polymeric nanoparticles during degradation can be tracked, providing insights into the rate and nature of the process. Furthermore, changes in dissipation may suggest potential modifications to the structure of the polymeric nanoparticles due to degradation. In this experiment, Au chips were functionalized with PDMAEMA, similar to the Si substrates, and the SC-PNPs were deposited in the QCM chamber. After the rinsing process, the surface density by the particles was confirmed and verified using AFM (Figure 8, Figures S2 and S3) before the actual degradation was investigated. The QCM-D degradation measurement curves are presented in Figure 9. At the beginning of the curves, there is a sharp increase in mass and dissipation for all measurements. This behavior was expected and can be interpreted as the binding of proteinase K to the SC-PNP. The increasing dissipation indicates a higher damping effect, and the additional enzyme layer increases the viscoelasticity of the samples. This suggests that proteinase K is softer than the polymer. Shortly after, there is a rapid decrease in mass and dissipation until the curves stabilize, with only slight changes in mass and dissipation after approximately ten minutes. The rapid loss of mass within the first ten minutes after the peak of enzyme binding most likely represents the degradation of the amorphous regions [10,11,14]. This is also reflected in the decreasing dissipation, which corresponds to a stiffening layer with diminishing damping properties [34] and indicates an increasing relative crystallinity fraction by reducing the relative amorphous fraction of the particles [35]. Such a decline is not observed in SC-PNPs without EtGly, as they contain fewer amorphous regions that are also more effectively shielded by the crystalline regions [35,36]. Furthermore, it can be observed that a larger initial increase in mass leads to a steeper decline in mass afterwards. This means that an increased binding of proteinase K enhances the degradation rate. The potential enhancement of enzyme binding due to a higher EtGly content is due to the ester linkages, which are more accessible due to the lower crystalline content. Proteinase K can bind to these ester linkages [9,37]. The relationship between EtGly content, enzyme binding, and degradation rates is summarized in Table 3. The first column in the “at the start” degradation rate pertains to the initial five minutes following the measurement of the maximum deposited mass. Meanwhile, the second column in the “at the end” degradation rate assesses the final ten minutes of the curves, where only a minimal decrease is observed. The increase in mass that was measured corresponds to the enzyme deposited. It is plausible that the maximum measured mass is smaller than the total enzyme mass deposited, given that degradation commences concurrently with enzyme binding. After the first ten minutes, degradation appears to proceed at a very slow pace, which explains why SC-PNP particles either maintain their size or experience only minimal size reduction after degradation. It can be explained that the amorphous regions degrade rapidly, leaving behind a stable crystalline core composed of a SC that acts as a resilient framework within the particle, resisting hydrolysis. Another plausible explanation for the slowed degradation may be the decreasing accessibility of the PlLA chains during the degradation process. Ultimately, after a sufficiently prolonged degradation period, the assumed crystalline framework within the SC-PNP particles disintegrates, resulting in the complete degradation of the particle.

#### 3.1.4. Degradation Evaluation of PNPs without Stereocomplexation

**Figure 10:**
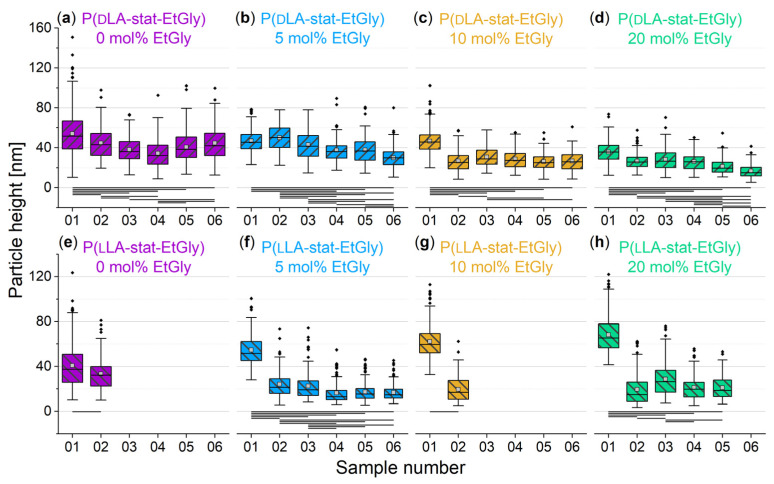
PNP particle heights without stereocomplexation determined via AFM before and after the degradation. (**a**–**d**) PdLA-PNPs, (**e**–**h**) PlLA-PNPs. Lines below bars indicate statistical differences in particle height in the group (*p* < 0.05).

As a control experiment, the degradation of PNPs without stereocomplexation, i.e., pure PlLA or PdLA with the same EtGly contents, was also investigated. To avoid the influence of different starting conditions on different substrates, only one sample was prepared for each case and the degradation was interrupted at the corresponding time points. However, the sample surface was still examined in a sampling manner, ensuring that 100 particles were measured to consider the measurement representative for the entire sample. The evaluation of particle heights during the degradation of all PNPs is shown in Figure 10. An exemplary AFM images before and after the degradation of PNPs are presented in Figure S4.

When compared with Figure 10a–d, representing the PdLA-PNP data, we notice that the particles have experienced a slight reduction in size due to degradation. However, in some instances, especially in Figure 10a for the PdLA-PNP without EtGly, there is a subsequent increase in particle height with prolonged degradation, albeit within the same range of fluctuations observed for the SC-PNPs. It is unlikely that this signifies an actual size increase due to particle swelling. Hence, it is reasonable to assume that the PdLA-PNPs without EtGly (Figure 10a) have undergone only minimal size reduction. This is further supported by the absence of a statistically significant difference in average particle heights between 1.5 and 24 h of degradation. Conversely, with the presence of EtGly in PdLA-PNPs (Figure 10b–d), the particles exhibit a more significant size reduction, which continues with extended degradation times. This is in contrast to PdLA-PNPs without EtGly, where the particle height began to increase again. Notably, in all cases, a decrease in size is evident, and this reduction is most pronounced within the initial 1.5 h. Since pure PdLA is biostable, it is presumed that the EtGly-based ester moieties in the particles undergo hydrolysis, leading to a decrease in particle size. Turning to Figure 10e–h, which depict PlLA-PNP data, it is worth noting that experiments have not been completed for all samples, primarily due to the absence of PNPs on some substrates after specific degradation times. Examining particle heights, a significant size reduction in PlLA-PNPs is observed after the initial 1.5 h of degradation for all samples. In the two cases where further measurements were possible (Figure 10f,h), the size appeared to stabilize thereafter. This suggests that, similar to PdLA-PNPs, a stable size has been reached for the PlLA-PNPs.

**Figure 11:**
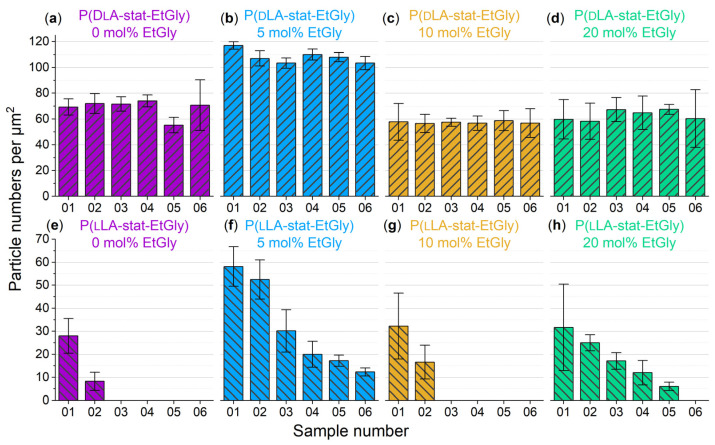
Area density of the PNPs without stereocomplexation before and after degradation. (**a**–**d**) PdLA-PNPs, (**e**–**h**) PlLA-PNPs.

In addition to particle heights, we also considered the surface density of the substrates covered by the PNPs. The assessment of surface densities for all samples during degradation is presented in Figure 11. For samples containing PdLA-PNPs (Figure 11a–d), the surface density displays only minimal fluctuations, irrespective of the EtGly content, and remains generally constant. This supports the assumption of biostable particles that do not undergo significant degradation, aligning with the existing literature findings [9]. For the evolution of surface density for PlLA-PNPs during degradation (Figure 11e–h), a noticeable decrease in particle count is observed with increasing degradation times across all samples. Notably, the second sample of PlLA-PNPs, with a 5 mol% EtGly content, exhibits the highest particle coverage. This likely explains why particles were still present on this sample even after 24 h. Initially, the coverage on the other three samples was approximately consistent, around 30 particles per µm^2^. When comparing the extent of the decrease, it becomes evident that the reduction in surface density is impeded with higher EtGly contents. Whereas pure PlLA is known to be rapidly hydrolyzed, the presence of EtGly suggests that the polymer PEtGly is somewhat more stable against hydrolysis compared to PlLA [9,26,29]. However, it should be noted that, based on our observations, it is not fully biostable like pure PdLA.

**Figure 12:**
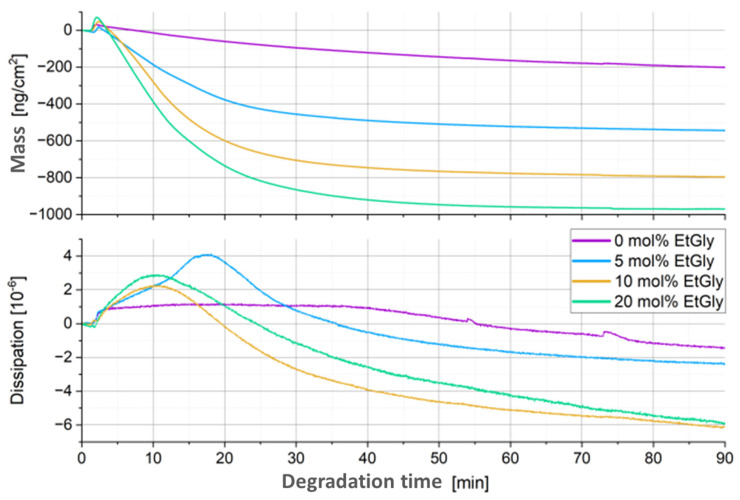
QCM-D measurement curves of the degradation of PlLA-PNPs.

To further verify this, QCM-D measurements were conducted for the PlLA-PNPs. The procedure was the same as for the SC-PNPs, and the coverage of the Au chips was also confirmed using AFM. Furthermore, this additional experiment aimed to provide better insight into the prematurely terminated experiment of the PlLA-PNPs with 10 mol% EtGly. The results of the QCM-D measurements are shown in Figure 12. Here as well, the attachment of proteinase K is observed as an increase in mass and dissipation in the first few minutes [26,34]. Subsequently, the mass decreases, and the total mass loss is much greater than in the degradation of the SC-PNPs. Furthermore, the overall mass loss is greater with a higher EtGly content in the PlLA-PNPs. Increased enzyme attachment is also observed with a higher EtGly content, which can explain the greater mass loss. Table 4 provides an overview of enzyme attachment and degradation rates, which are similar to Table 3. The maximum measured masses are in the same order of magnitude as those of the SC-PNP (Table 4). However, the degradation rates of the PlLA-PNPs are much higher (especially at the beginning) than those for the SC-PNPs. It is clearly noticeable that at the beginning (first column of the degradation rates—the next five minutes after the maximum measured mass), the rate is higher with a higher EtGly content or enzyme attachment. In the last ten minutes of the measurement, this trend completely reverses, indicating that the faster degradation process observed at the beginning is likely completed earlier. Contrary to the observations made using AFM, it appears that the EtGly content does not inhibit but rather accelerates degradation. When examining the trend of dissipation, it also seems that the EtGly content has an influence on the strength of the particles during degradation. In the case of pure PlLA-PNPs without EtGly, the increase and subsequent decrease in dissipation are minimal compared to the trend observed in all PlLA-PNPs with higher EtGly contents. Therefore, a PlLA-PNP with a higher EtGly content should exhibit greater stiffness after degradation compared to before degradation and even compared to the pure PlLA-PNPs without EtGly.

### 3.2. Influence of the SC, the Crystallinity, and the EtGly Amount on the Properties

To understand the impact of the EtGly content and stereocomplexation, along with its associated crystallinity, during degradation, we first determined the initial mechanical properties such as the elastic modulus. We accomplished this by analyzing force–distance curves (FDCs) to establish the elastic modulus for all SC-PNPs and PNPs without stereocomplexation.

**Figure 13:**
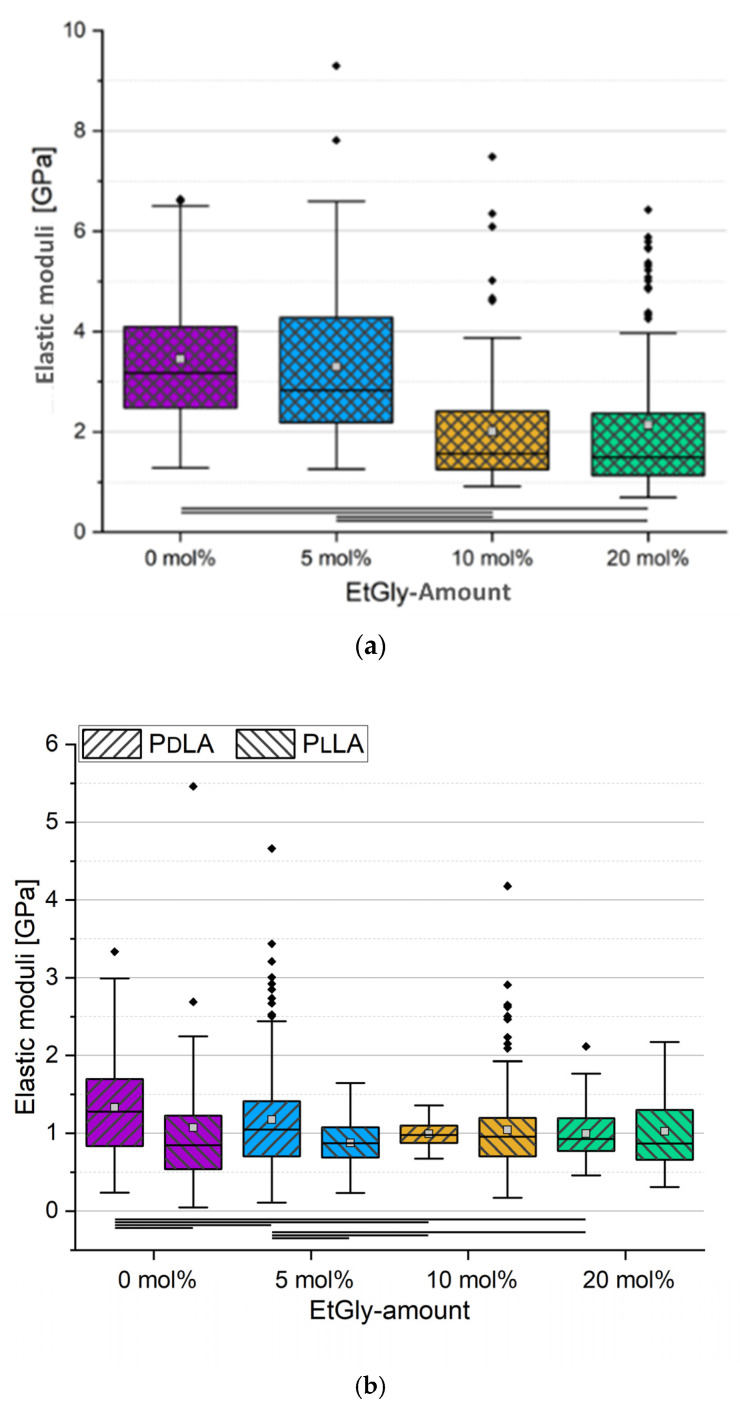
Elastic moduli of all SC-PNPs (**a**) and all PNPs without stereocomplexation (**b**) determined using FDCs. Lines below bars indicate statistical differences in elastic moduli in the group (*p* < 0.05).

The results are presented in the box plots displayed in Figure 13a,b. A representative measurement curve is provided in Figure S5. For SC-PNPs, a clear trend emerges: a higher EtGly content correlates with a reduced elastic modulus. This aligns with the theory [18] that EtGly introduces steric hindrance into the polymer, hindering the stereocomplexation process. Consequently, the nanoprecipitation yields more amorphous and less crystalline components, resulting in softer SC-PNPs with a higher EtGly content. Whereas not all samples displayed statistically significant differences, the overall trend appears linear, especially when considering the median line. The mean values of the SC-PNPs with 5 and 20 mol% EtGly contents is influenced by outliers, pulling them upward. In the case of PNPs without stereocomplexation, we compared PdLA-PNP and PlLA-PNP samples and also assessed differences within PNPs with the same EtGly contents. Statistically significant distinctions were primarily observed in PNPs with low EtGly contents. It appears that PdLA-PNPs with low EtGly contents tend to be stiffer, but this difference diminishes as the EtGly content increases.

Comparing SC-PNPs with PNPs without stereocomplexation in terms of their modulus of elasticity reveals a consistent trend: SC-PNPs consistently exhibit higher values. This higher modulus in the SC-PNPs results from increased crystallinity due to stereocomplexation, which is in line with previous research findings [18,24,38]. As crystallinity decreases with higher EtGly contents, the modulus of elasticity of the SC-PNPs approaches that of PNPs without stereocomplexation. PNPs without stereocomplexation are assumed to be entirely amorphous, which explains why even SC-PNPs with 20 mol% EtGly content still, on average, exhibit a higher modulus of elasticity [24]. However, it is important to note that SC-PNPs have a larger diameter and a greater particle height than PNPs without stereocomplexation, which may have influenced the measurementsof PdLA-PNPs and PlLA-PNPs, potentially causing the values of PNPs without stereocomplexation to be shifted upwards [18,38,39]. 

After degradation, further analysis of FDCs was not possible in any case. The recorded curves of the SC-PNPs degraded for 24 h exhibited an atypical behavior that did not allow for analysis. Exemplary curves are shown in Figure S6. In both cases, it can be observed that the approach curves (blue) do not follow a linear trend but move almost vertically upwards. Upon closer inspection, small deviations can be seen in the zoomed-in sections inserted in the top right corner of each graph. Since this behavior was consistently observed in almost all particle measurements, while the curves on the substrate followed the expected pattern, it can be concluded that this is not a measurement artifact. The deviation in the curve can be explained by the breakthrough of the AFM cantilever through the particles. It is suspected that after degradation of the SC-PNP, only a framework of the crystalline regions of the particle remains. This framework appears to yield and collapse under the pressure exerted by the cantilever during the measurement, causing the cantilever to snap back multiple times. Subsequently, the cantilever bends again. This sequence of events leads to the atypical curve shapes shown in Figure S6, with the described deviations [40]. For the degraded PNPs without stereocomplexation, it was not possible to perform FDC measurements in general. As expected, the PlLA-PNPs degraded, resulting in a lack of sufficient particles remaining on the substrates for conducting measurements. The PdLA-PNPs without EtGly exhibited biostability, making a second round of FDC measurements for this sample redundant. After degradation, all other PdLA-PNPs had average heights of less than 30 nm, which is insufficient for FDC measurements. Hence, no measurements were conducted in these cases as well.

## 4. Conclusions

We conducted a comprehensive investigation into the degradation behavior of SC-PNPs, exploring their response to varying EtGly proportions and their influence on crystallinity. AFM did not reveal any changes within the first 24 h but indicated accelerated degradation after 7 days when a higher EtGly content was present, implying that lower crystallinity renders the particles more susceptible to hydrolysis. QCM-D exhibited reduced enzyme adsorption and a slower degradation rate in SC-PNPs with lower EtGly contents and higher crystallinities. A more in-depth analysis of the degradation process unveiled that QCM-D detected rapid degradation from the outset, whereas AFM exhibited delayed changes in degradation. This degradation mechanism can be described as volume erosion, characterized by minimal size alterations but a diminishing PNP surface coverage [33]. SC-PNPs disintegrated gradually without changes in size, primarily within the amorphous regions of the polymer, whereas the crystalline framework decayed more slowly until complete dissolution [33,36,38]. As a control experiment, we investigated the degradation of PlLA-PNPs and PdLA-PNPs without stereocomplexation. Our findings corroborated the existing literature for PNP films: PdLA-PNPs displayed robust biostability, whereas PlLA-PNPs degraded rapidly [9]. Although EtGly demonstrated susceptibility to hydrolysis, we have not been able to establish a definite influence for EtGly on PNP degradation without stereocomplexation at this stage. SC formation led to the formation of larger PNPs under identical conditions and conferred greater stiffness, particularly when some crystallinity was present. As the EtGly content increased and the crystallinity decreased, the modulus of elasticity of the SC-PNPs approached that of PNPs without stereocomplexation [18,24,38].

In summary, our study has established the groundwork for future research on SC-PNPs derived from PdLA and PlLA. Although we have clarified the degradation mechanism, further investigation is needed to ascertain its effects on drug release and its practical applications in drug delivery. Delving into these aspects will offer a comprehensive insight into the potential utility of these particles in the field of drug delivery.

## Figures and Tables

**Table 1:** Selected characterization data of the synthesized (co)polymers. Molar masses and dispersity values *Ð* were determined by size exclusion chromatography using refractive index detection and polystyrene calibration [20].

Polymer	mol% LA/EtGly	*M_n_* [kg mol^−1^] SEC^3^	*Đ* SEC^3^
PlLA	100/0	16	1.06
PdLA	100/0	19	1.06
P(lLA-*stat*-EtGly)	95/05	16	1.11
P(lLA-*stat*-EtGly)	90/10	13	1.20
P(lLA-*stat*-EtGly)	80/20	12	1.23
P(dLA-*stat*-EtGly)	95/05	19	1.10
P(dLA-*stat*-EtGly)	90/10	18	1.21
P(dLA-*stat*-EtGly)	80/20	15	1.28

**Table 2:** Summary of the degradation time of the individual samples available for all EtGly amounts.

**Sample number**	01	02	03	04	05	06
**Degradation time [h]**	0.0	1.5	3.0	4.5	6.0	24.0

**Table 3:** Maximum deposition mass and degradation rates determined via QCM-D of the SC-PNPs.

EtGly Amount	$\mathrm{Maximum}\;\mathrm{Accumulated}\;\mathrm{Mass}\;[\frac{ng}{{cm}^{2}}]$	$\mathrm{Degradation}\;\mathrm{Rate}\;[\frac{ng}{{cm}^{2}min}]$	
		At the Beginning	At the End
0 mol%	11.6	−0.37	−0.12
5 mol%	20.3	−1.33	−0.21
10 mol%	66.1	−8.55	−0.34
20 mol%	46.3	−3.44	−0.18

**Table 4:** Maximum deposition mass and degradation rates determined via QCM-D of the PlLA-PNPs.

EtGly Amount	$\mathrm{Maximum}\;\mathrm{Accumulated}\;\mathrm{Mass}\;[\frac{ng}{{cm}^{2}}]$	$\mathrm{Degradation}\;\mathrm{Rate}\;[\frac{ng}{{cm}^{2}min}]$	
		At the Beginning	At the End
0 mol%	30.9	−5.76	−1.14
5 mol%	16.7	−26.21	−0.66
10 mol%	47.1	−39.17	−0.46
20 mol%	70.5	−57.80	−0.10

## Data Availability

Data are contained within the article and Supplementary Materials.

## Supplementary Materials

The following supporting information can be downloaded at: https://www.mdpi.com/article/10.3390/nano14050440/s1. Figure S1. Representative AFM images of the SC nano particles (with 20% EtGlty) before (left) and after (right) the 7 days degradation. Figure S2. Representative AFM images of the functionalized Au chips with SC-PNP deposited on them after the rinsing process but before the degradation. Figure S3. Representative AFM images of the functionalized Au chips with PlLA-PNP after the rinsing process but before degradation. Figure S4. Representative AFM images of the nanoparticles before (left) and after (right) degradation (10 mol% EtGly, after 4.5 days of degradation). Figure S5. Representative FDC measurement force-distance curve of a SC-PNP with 5 mol% EtGly. Figure S6. Representative curves of recorded FDCs of SC-PNP after 24 h degradation.
