# Supplementary material for: The Effect of Stereocomplexation and Crystallinity on the Degradation of Polylactide Nanoparticles
Source: Nanomaterials (Basel). 2024 Feb 28;14(5):440. doi: 10.3390/nano14050440 (PMC10934930; doi:10.3390/nano14050440)
Supplement: Supplementary file 1 [file nanomaterials-14-00440-s001.zip › nanomaterials-2832395-supplementary.pdf]

nanomaterials

Article

The Effect of Stereocomplexation and Crystallinity on the Degradation of Polylactide Nanoparticles

Chuan Yin [1,†], Jenny Hemstedt [1,†], Karl Scheuer [1], Maja Struczyńska [1,2], Christine Weber [3], Ulrich S. Schubert [3], Jörg Bossert [1] and Klaus D. Jandt [1,2,*]

[1] Chair of Material Science (CMS), Otto Schott Institute for Materials Research (OSIM), Friedrich Schiller University Jena, Löbdergraben 32, 07743 Jena, Germany; chuan.yin@uni-jena.de (C.Y.); hemstedt99@gmail.com (J.H.); karlscheuer31@gmail.com (K.S.); maja.struczynska@uni-jena.de (M.S.); joerg.bossert@uni-jena.de (J.B.)
[2] Jena School for Microbial Communication (JSMC), Neugasse 23, 07743 Jena, Germany
[3] Institute of Organic Chemistry and Macromolecular Chemistry (IOMC), Friedrich Schiller University Jena, Humboldtstrasse 10, 07743 Jena, Germany; christine.weber@uni-jena.de (C.W.); ulrich.schubert@uni-jena.de (U.S.S.)
* Correspondence: Author: k.jandt@uni-jena.de
† These authors contributed equally to this work.

Supplementary materials

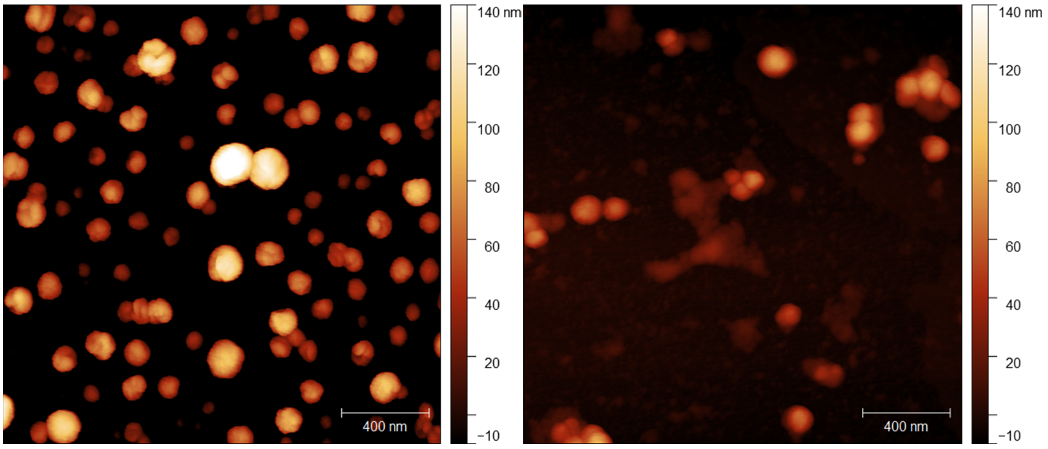

Figure S1. Representative AFM images of the SC nano particles (with 20% EtGlty) before (left) and after (right) the 7 days degradation.

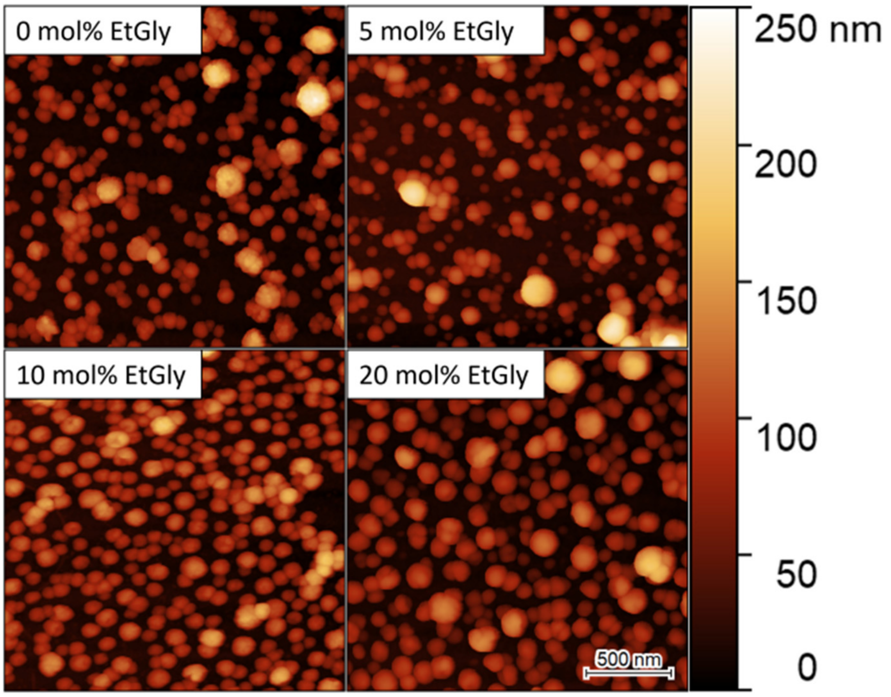

Figure S2. Representative AFM images of the functionalized Au chips with SC-PNP deposited on them after the rinsing process but before the degradation.

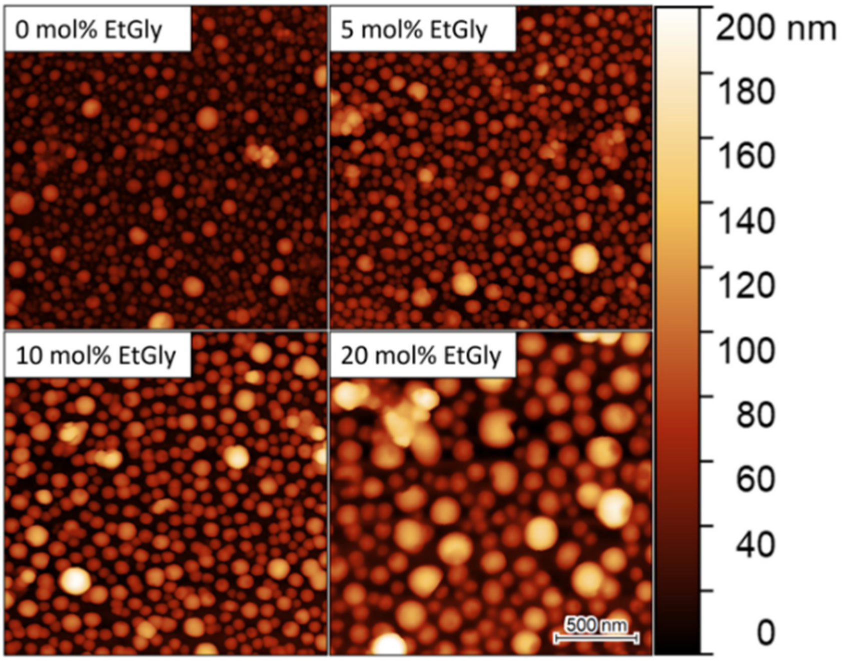

Figure S3. Representative AFM images of the functionalized Au chips with PLLA-PNP after the rinsing process but before degradation.

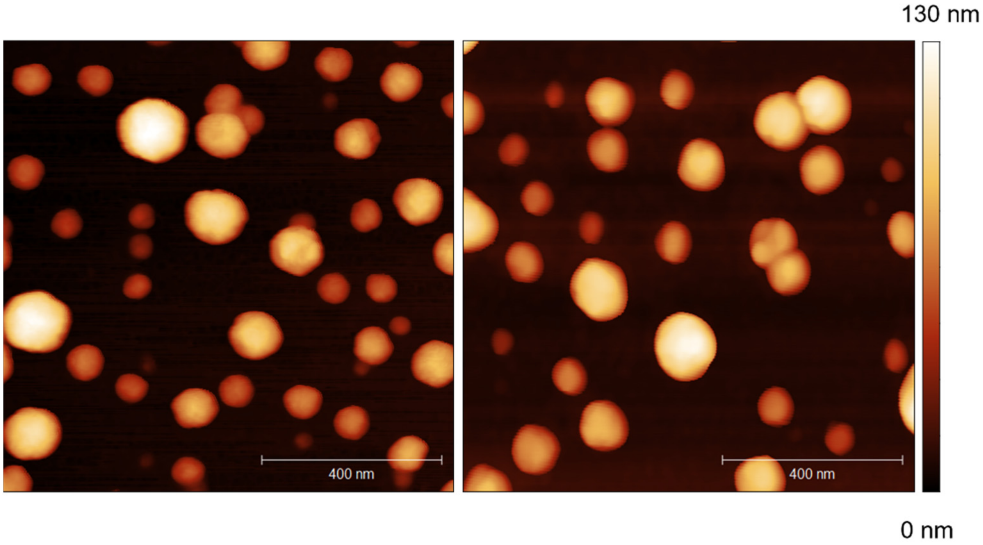

Figure S4. Representative AFM images of the nanoparticles before (left) and after (right) degradation (10 mol% EtGly, after 4.5 days of degradation).

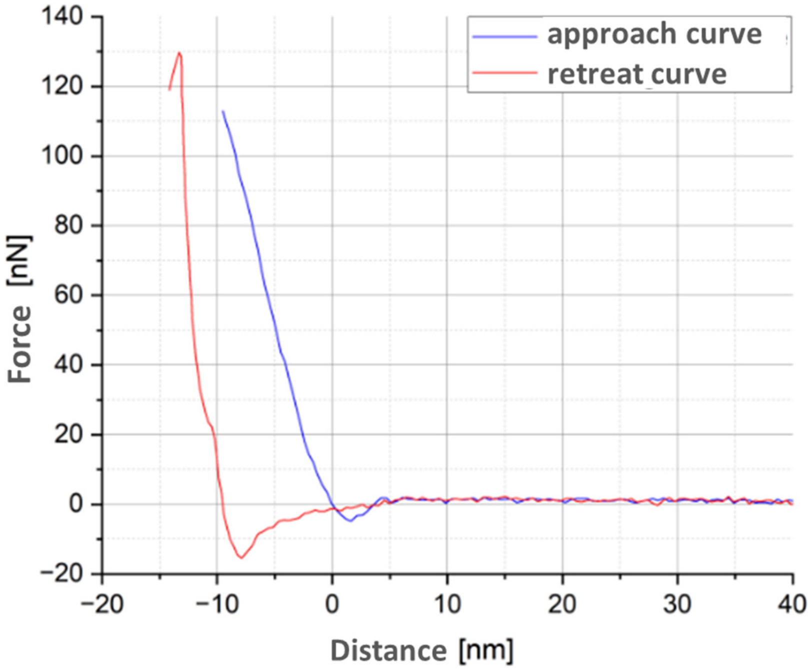

Figure S5. Representative FDC measurement force-distance curve of a SC-PNP with 5 mol% EtGly.

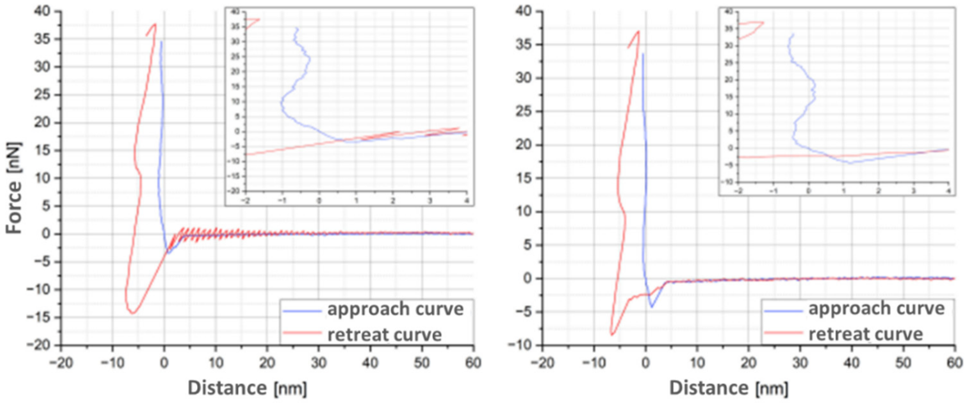

Figure S6. Representative curves of recorded FDCs of SC-PNP after 24 hours degradation.
